# Multisystemic Therapy and Functional Family Therapy Compared on their Effectiveness Using the Propensity Score Method

**DOI:** 10.1007/s10802-017-0392-4

**Published:** 2018-01-09

**Authors:** Hester V. Eeren, Lucas M. A. Goossens, Ron H. J. Scholte, Jan J. V. Busschbach, Rachel E. A. van der Rijken

**Affiliations:** 1 Viersprong Institute for Studies on Personality Disorders (VISPD), Halsteren, The Netherlands; 2 Department of Psychiatry, Section Medical Psychology and Psychotherapy, Erasmus Medical Center, Rotterdam, The Netherlands; 3 Erasmus School of Health Policy and Management, Erasmus University Rotterdam, Rotterdam, The Netherlands; 4 Behavioural Science Institute, Radboud University Nijmegen, Nijmegen, The Netherlands

**Keywords:** Propensity score, Comparative effectiveness research, Adolescent, Quasi-experimental study, Behavioral problems

## Abstract

Multisystemic Therapy (MST) and Functional Family Therapy (FFT) have overlapping target populations and treatment goals. In this study, these interventions were compared on their effectiveness using a quasi-experimental design. Between October, 2009 and June, 2014, outcome data were collected from 697 adolescents (mean age 15.3 (*SD* 1.48), 61.9% male) assigned to either MST or FFT (422 MST; 275 FFT). Data were gathered during Routine Outcome Monitoring. The primary outcome was externalizing problem behavior (Child Behavior Checklist and Youth Self Report). Secondary outcomes were the proportion of adolescents living at home, engaged in school or work, and who lacked police contact during treatment. Because of the non-random assignment, a propensity score method was used to control for observed pre-treatment differences. Because the risk-need-responsivity (RNR) model guided treatment assignment, effectiveness was also estimated in youth with and without a court order as an indicator of their risk level. Looking at the whole sample, no difference in effect was found with regard to externalizing problems. For adolescents without a court order, effects on externalizing problems were larger after MST. Because many more adolescents with a court order were assigned to MST compared to FFT, the propensity score method could not balance the treatment groups in this subsample. In conclusion, few differences between MST and FFT were found. In line with the RNR model, higher risk adolescents were assigned to the more intensive treatment, namely MST. In the group with lower risk adolescents, this more intensive treatment was more effective in reducing externalizing problems.

**Electronic supplementary material:**

The online version of this article (10.1007/s10802-017-0392-4) contains supplementary material, which is available to authorized users.

Multisystemic Therapy (MST) and Functional Family Therapy (FFT) both originated from the United States (US). Their proven effectiveness in reducing adolescents’ antisocial behavior and delinquency has led to the worldwide dissemination of these interventions. Both MST and FFT are aimed at reducing the behavioral problems of 12–18 year old adolescents by intervening in the youth’s family and environmental system. Functional Family Therapy has an integrated theoretical base in which behavioral techniques, system perspectives, and cognitive theory are combined while remaining informed by intrapsychic perspectives (Breuk et al. 2006; Sexton and Alexander 2003). Antisocial behavior is thought to be mediated and embedded in a complex sequence of relations between the adolescent and his or her family members (Sexton and Alexander 2003). Therefore, FFT is specifically aimed at improving family communication and supportiveness while decreasing negativity and dysfunctional behavioral patterns (Blueprints for healthy youth development 2015). The therapy mainly consists of direct contact with family members, but may be coupled with support system services, such as school or work. Research has shown that FFT is effective in reducing (delinquent) behavioral problems, recidivism, and substance abuse, and that it guides family members in improving their family situation (Alexander and Sexton 2002; Sexton and Alexander 2000; Sexton and Turner 2010).

Caregivers are also seen as the most important link in the treatment process of MST, but MST also actively involves all other systems surrounding the youth, such as friends, schools, and neighborhoods (Henggeler et al. 2009). This approach is founded in the social-ecological theory of Bronfenbrenner (1979), in which it is thought that antisocial behavior is multi-determined by the different social systems in which an individual acts. By intervening in and with these social systems, risk factors are reduced and a youth’s social environment is changed such that it stimulates prosocial activities instead of antisocial behavior (Henggeler et al. 2009). Multisystemic Therapy is more intensive than FFT, because a therapist visits the family at home and is available to the family round-the-clock. Research has shown that MST effectively reduces behavioral problems and delinquency, recidivism, substance abuse, out-of-home placement, family problems, and involvement with deviant peers (Henggeler 2011; van der Stouwe et al. 2014).

The effectiveness of both MST and FFT is well-established compared to regular treatment, such as individual treatment, family-based interventions, or parenting counseling (Asscher et al. 2013; Sundell et al. 2008). Multisystemic Therapy and FFT clearly show overlap in their target populations and treatment goals (e.g., Chorpita et al. 2011; Henggeler 2011; Sexton and Turner 2010). Given this overlap, the question arises what intervention works best for whom. However, little is known about their relative effectiveness (i.e., whether one intervention outperforms the other).

A recent study by Baglivio et al. (2014) compared the effectiveness of MST and FFT in juvenile practice in the US. In this study, youth receiving MST or FFT had been referred by probation officers from the juvenile justice department. Results showed little significant difference in the effectiveness of the two interventions. However, low-risk youth receiving FFT committed fewer offenses during treatment than low-risk youth receiving MST. Because referral practices and treatment populations differ between countries (Asscher et al. 2013; Sundell et al. 2008), the relative effectiveness of MST and FFT is unknown outside the US. In the Netherlands, youth are referred to MST or FFT by various referral agencies, including the Child Protection Council, juvenile judges, local referral institutions, and primary health care providers. Compared to allocation procedures in the US, in the Netherlands youth are less often referred to MST or FFT by a judicial agency. This could influence the target population as well as treatment effects. We, therefore, studied the relative effectiveness of these interventions in the Netherlands.

To allocate adolescents and their families to either one of the treatments, a well-known model, the Risk-Need-Responsivity (RNR) model, is often used. Following this model, the intensity of the treatment should be matched to risks and characteristics of the adolescent. The higher the risk of delinquent behavior, the more intensive treatment should be (Andrews and Bonta 2010; Andrews et al. 2006). The model implies that adolescents should be assigned to FFT unless there are indications that MST would be more suitable, such as serious delinquent behavior, a high risk that the family cannot provide a safe environment, and an increased risk of recidivism (Oudhof et al. 2009). In practice, this assignment procedure is followed by clinicians assigning youth to either FFT or MST. A previous Dutch study comparing both treatment populations found that more youth receiving MST had a court order than youth receiving FFT, and that youth receiving MST had more risk factors than those receiving FFT (Hendriks et al. 2014). This finding is in accordance with the results of a Swedish study which demonstrated that youth receiving MST had more behavioral problems than youth receiving FFT (Gustle et al. 2006). However, although both European studies showed that the most at risk youth received the most intensive treatment (i.e., MST), the model leaves room for interpretation and may be subject to chance. In fact, the target populations of MST and FFT show substantial overlap (Hendriks et al. 2014). Therefore, it appears that criteria used to allocate adolescents and their families to either one of the treatments are not fully mutually exclusive. Because these studies only looked into treatment populations and did not consider treatment effects, it remains unknown which intervention is the most effective for these overlapping target populations.

Therefore, the current study aimed to investigate the relative effectiveness of MST and FFT in the Netherlands. Because interventions are compared in their everyday practice settings, a quasi-experimental design was used, meaning that youth were not randomly allocated to one of the interventions. Without controlling for pre-treatment differences, a difference in outcomes may either be caused by the intervention itself, or by pre-treatment characteristics of adolescents and their families. Therefore, a propensity score (PS) was estimated and used to control for this ‘allocation bias’.

The use of a PS in psychological research has increased in recent years (e.g., Austin 2011; Green and Stuart 2014; Thoemmes and Kim 2011; West et al. 2014). The current study used these tutorials and literature as a starting point in comparing MST and FFT. Because previous research has shown that youth receiving MST were more at risk than youth receiving FFT (Gustle et al. 2006; Hendriks et al. 2014), and because the only study to directly compare the effectiveness of FFT and MST thus far has taken risk level into account as well (Baglivio et al. 2014), the current study compared the treatment effects not only for the whole sample, but also in two subsamples of youth: with and without a court order. Having a court order can be interpreted as a risk factor and indicate the risk level of an adolescent before treatment. Based on the RNR model, more youth without a court order would be expected to be referred to FFT than to MST and more youth with a court order would be expected to receive MST.

With a growing body of research examining evidence-based treatment, and given today’s stringent health care budgets, it seems only logical to allocate youth to a more intensive and likely more expensive treatment (i.e., MST) only when there is no effective alternative (i.e., FFT; Aos et al. 2004; Asscher et al. 2013; Vermeulen et al. 2017). By comparing evidence-based interventions, budget allocation and the assignment of youth to the right interventions can be optimized.

## Methods

### Participants

Because the assignment procedure following the RNR model implies that adolescents should be assigned to FFT unless there are indicators that MST would be more suitable (Oudhof et al. 2009), FFT was considered the reference treatment and MST the ‘new’ treatment. Between October, 2009 and June, 2014, 1714 adolescents and their families started either FFT (*N* = 640) or MST (*N* = 1074) at De Viersprong, institute for personality disorders and behavioral problems in the Netherlands. After finishing treatment, 697 (40.7%) participants completed the primary outcome measure (i.e., the Child Behavior Checklist, CBCL). These were 275 (43%) adolescents who had received FFT and 422 (39.3%) adolescents who had received MST. Such a low percentage of completed questionnaires after treatment is not uncommon within Routine Outcome Monitoring (ROM) because data is not gathered for specific research purposes (Stichting Benchmark GGZ 2016). To reduce uncertainty in the statistical analyses and results, these 697 families formed the study sample for the statistical analyses. Adolescents who had received FFT and completed the primary outcome measure differed significantly from those who did not with regard to their country of birth, living situation, and whether or not they had a court order before treatment (see Table I in Online Supplemental Material). Adolescents who received MST and completed the assessment after finishing treatment differed from those who did not with regard to their country of birth, living situation, engagement in school or work, whether or not they had a court order before treatment, as well as the country of birth, level of education, and employment status of their primary caregiver, and whether or not this primary caregiver had a partner (see Table II in Online Supplemental Material).

In addition to the study sample of 697 adolescents, the effectiveness of the treatments was compared between the two subsamples of youth with and without a court order. Of the 422 adolescents who received MST, 246 had a court order and 168 had not (for 10 adolescents [2 FFT; 8 MST], the judicial status was unknown). For FFT, 71 adolescents had a court order, while 202 had not.

### Procedures

As part of the treatment procedure, adolescents and their families filled in questionnaires for ROM at the beginning of and after completing treatment. Routine Outcome Monitoring is a measurement system to routinely collect data on the outcome of treatment, evaluate individual treatment progress, and provide transparency regarding the effectiveness of treatment (Buwalda et al. 2011). Within ROM, adolescents and their families provide consent concerning the collection of data and its use for quality control and research. The Medical Ethical Committee of the Erasmus Medical Centre approved this study (METC-2015-124).The quality of treatment delivery of MST and FFT was monitored by the quality assurance systems of both interventions. These systems provide guidelines for therapist training and supervision, adherence to the treatment protocol, and treatment duration. In the current study, all therapists were trained and licensed to deliver the treatments. According to the treatment model, they received weekly supervision from their team supervisor and from an independent consultant (i.e., working for the licensor of MST or FFT instead of the provider organization itself). Data on treatment adherence and treatment duration were obtained from MST Institute and FFT LLC, who manage the quality assurance systems of MST and FFT, respectively. Within the study sample, the mean treatment duration of MST was 148.5 days, which was somewhat longer than the expected duration of 100 to 140 days according to the MST quality assurance system. For FFT, the mean treatment duration was 196.2 days, which was also above the expected duration (i.e., 90 to 150 days according to the FFT quality assurance system). Both MST and FFT therapists were adherent to the treatment model, meaning they were consistently implementing the model with their cases. The mean adherence score within MST was .53, which was in accordance with the norm of ≥0.50 provided by MST Institute. Within FFT, the mean adherence score was 3.97, which was above the FFT LLC target of 3.0.

### Measures

#### Baseline Measures

##### Demographics

To correct for initial differences between the treatment groups, an extensive set of questionnaires was completed at the beginning of the treatment. The therapist reported several demographics of the adolescents and their primary caregivers. Age, gender, country of birth, living situation, level of education, previous treatment, engagement in school or work, previous court orders, police contacts, and the relation with their father, mother, siblings, and peers were reported for each adolescent. Furthermore, the country of birth, level of education, employment status, and presence of a partner were reported for the primary caregivers (Praktikon/MST-NL, Sociaal Demografische Informatie. Ongepubliceerde vragenlijst [Demographic information], unpublished manuscript). Table 1 shows all demographic characteristics at baseline for both treatment groups.

**Table 1:** Baseline differences between adolescents assigned to MST and FFT and standardized bias in full sample (*N* = 697)

Variable		FFT	*(n = 275)*		MST	*(n = 422)*		Test statistic	Standardized bias	
Continuous variables		Mean	SD	N	Mean	SD	N	T-test	Before PS application	After PS application
Age		15.9	1.59	275	15.67	1.35	422	1.96	0.17	0.12
CBCL	Internalizing problems	62.51	9.26	263	61.04	9.68	409	1.95	0.15	0.01
*Primary outcome*	Externalizing problems	67.08	9.57	263	68.29	10.06	409	−1.56	0.12	0.07
	Total behavioral problems †	66.04	8.61	263	65.32	9.76	409	1.00	0.07	0.01
YSR	Internalizing problems	54.79	11.31	246	50.78	11.5	356	4.24***	0.35	0.11
	Externalizing problems	59.27	9.73	246	57.54	10.87	356	2.04*	0.16	0.04
	Total behavioral problems	57.35	9.78	246	53.59	11.02	356	4.40***	0.34	0.07
Parenting stress		1.97	1.78	258	2.06	2.07	397	−0.61	0.05	0.06
Categorical variables		%		N	%		N	Chi-Square statistic		
Gender	Male	53.6		141	67.2		275	12.60***	0.29	0.23
	Female	46.4		122	32.8		134		0.29	0.23
Country of birth	Netherlands	95.8		253	83.4		341	24.04***	0.19	0.06
	Western country	1.1		3	4.6		19		0.05	0.04
	Non-Western country	3.0		8	12.0		49		0.13	0.02
Living situation adolescent	Together with one parent	36.1		97	42.9		179	6.93*	0.12	0.06
	Together with multiple parents	60.6		163	51.1		213		0.16	0.04
	Other	3.3		9	6.0		25		0.05	0.02
Living situation adolescent	Lived not at home	0.8		2	2.9		12	3.64	0.13	0.01
Secondary outcome	Lived at home	99.2		260	97.1		400		0.13	0.01
Level of education	None	7.1		19	13.7		56	32.55***	0.08	0.03
	Primary education	3.7		10	2.7		11		0.01	0.01
	Lower secondary education	54.5		146	66.8		274		0.15	0.11
	Higher secondary education	34.7		93	16.8		69		0.21	0.07
Previous treatment	Absent	9.8		26	5.5		23	4.46*	0.19	0.01
	Present	90.2		240	94.5		395		0.19	0.01
Engagement in school or work	Absent	14.5		37	22.6		91	6.61**	0.19	0.16
Secondary outcome	Present	85.5		219	77.4		312		0.19	0.16
Court order	No	74.0		202	40.6		168	75.91***	0.41	0.05
	Civil	10.6		29	30.9		128		0.25	0.18
	Criminal	15.4		42	28.5		118		0.16	0.13
Police contacts during treatment	Absent	66.9		176	50.8		198	16.74***	0.32	0.01
Secondary outcome	Present	33.1		87	49.2		192		0.32	0.01
Relation father	Absent	6.8		17	9.2		37	1.22	0.09	0.15
	Present	93.2		234	90.8		364		0.09	0.15
Relation mother	Absent	0.4		1	0.7		3	0.30	0.04	0.06
	Present	99.6		249	99.3		402		0.04	0.06
Relation siblings	Absent	7.6		18	6.0		23	0.64	0.07	0.02
	Present	92.4		218	94.0		361		0.07	0.02
Relation peers	Absent	0.0		0	1.3		5	3.15	0.11	0.00
	Present	100.0		249	98.7		393		0.11	0.00
Country of birth primary caregiver	the Netherlands	88.5		232	79.3		325	11.28**	0.13	0.02
	Western country	4.2		11	4.9		20		0.01	0.02
	Non-Western country	7.3		19	15.9		65		0.12	0.01
Level of education primary caregiver	None	1.2		3	3.0		12	8.17	0.02	0.05
	Primary education	4.1		10	8.0		32		0.04	0.00
	Lower secondary education	27.9		68	31.3		126		0.04	0.01
	Higher secondary education	45.5		111	39.1		157		0.07	0.04
	Higher education	21.3		52	18.7		75		0.03	0.02
Employment primary caregiver	Employed	71.8		186	61.9		253	6.98**	0.21	0.17
	Unemployed	28.2		73	38.1		156		0.21	0.17
Partner primary caregiver	Absent	21.7		55	23.9		94	0.41	0.05	0.01
	Present	78.3		198	76.1		299		0.05	0.01

* *p* < 0.05, ** *p* < 0.01, *** *p* < 0.001, † Not selected for PS estimation

Values depict the mean values and standard deviations. Except for age and parenting stress all other scores are standardized T-scores, having a mean of 50 and a standard deviation of 10. For NOSI-R and parenting stress, normed z-scores are displayed

MST, Multisystemic Therapy, FFT, Functional Family Therapy, CBCL, Child Behavior Checklist, YSR, Youth Self Report, SD, standard deviation, PS, propensity score

##### Problem Behavior

Furthermore, parents completed the CBCL (Achenbach and Rescorla 2001; Dutch version by Verhulst and van der Ende 2001a) and the youths themselves completed the Youth Self Report (YSR; Achenbach and Rescorla 2001; Dutch version by Verhulst and van der Ende 2001b). A youth’s internalizing problem behavior, externalizing problem behavior, and the total score of the problem behavior were used for analyses. On both questionnaires, items were completed on a 3-point scale (ranging from 0 = *never* to 2 = *often*). T-scores were computed and used for analyses. A higher T-score indicates that an adolescent has more problems. Both CBCL and YSR scales were used to measure problem behavior from different perspectives. The Cronbach’s alpha coefficients of the study sample for internalizing, externalizing, and total problem behavior measured with the CBCL were .88, .93, and .96, respectively. For the YSR these coefficients were .92, .90, and .95, respectively. The Cronbach’s alpha coefficients found in the study sample were similar to those reported in the CBCL and YSR manual (i.e., CBCL: .90, .94, and .97, YSR: .90, .90, and .95 Achenbach and Rescorla 2001).

##### Parenting Stress

Finally, until September, 2012, parenting stress was measured with the Nijmeegse Ouderlijke Stress Index (NOSI-R; De Brock et al. 2004) in which the primary caregiver completes 42 items on a 4-point scale (ranging from 1 = *fully disagree* to 4 = *fully agree*). These items are used to estimate a score for parenting stress wherein a higher score indicates more stress. The reliability coefficient was .95. From October, 2012 onwards, the Opvoedingsbelasting Vragenlijst (OBVL; Vermulst et al. 2012) was used to measure parenting stress. For this measure, the primary caregiver completes 34 items on a 4-point scale (ranging from 1 = *not true* to 4 = *very true*). For an English version of the OBVL, see http://www.praktikon.nl/wp-content/uploads/2016/03/obvl_en.pdf.

The scores of all items are summed for a total score regarding parenting stress. The alpha coefficient for this measure was .94. Because parenting stress was measured with two different questionnaires, the deviance score of the scales was used to express the level of parenting stress for both questionnaires in one score concerning parenting stress. This was estimated by subtracting the normscore from the score of the adolescent and dividing this by the standard deviation of the norm group.

##### Treatment Variables

Treatment variables, such as length of treatment and dosage of treatment, were not controlled for in the propensity score since these treatment characteristics are part of the treatment itself and the treatment is adapted to the specific situation of the adolescent and his or her family.

#### Outcome Measures

##### Primary Outcome Measure: Externalizing Problem Behavior

Because both FFT and MST are primarily aimed at reducing externalizing problem behavior, this was defined as the primary outcome measure and was measured with the CBCL and YSR (Achenbach and Rescorla 2001). The primary caregiver reported the externalizing problems of the adolescent with the CBCL, while the youth himself reported this behavior with the YSR. Both measures were completed at the start and the end of treatment by completing 35 items on a 3-point scale (ranging from 0 = *never* to 2 = *often*). T-scores were used for the analyses. A higher T-score indicates that an adolescent has more problems. The alpha reliability coefficient for the current sample at the end of the treatment using the CBCL and YSR is .94 and .88 respectively.

##### Secondary Outcome Measures

Three secondary outcome measures were assessed at the end of the treatment: 1) whether or not the youth was living at home (i.e., the adolescent had not been placed out of home); 2) whether or not the adolescent was engaged in school or work for at least 20 h per week at the end of the treatment; and 3) whether or not the adolescent had new police contact due to inappropriate or illegal behavior during the treatment period. The therapist registered these treatment outcomes after treatment and in consultation with the primary caregiver. These three outcomes have been operationalized and standardized by MST Services to ensure that these outcomes are scored identically by all therapists (MST Institute 2016). This scoring procedure was also followed by FFT. The quality assurance systems of both treatments ensure that their ultimate outcomes are monitored by the therapist, the team supervisor, and the team consultant.

### Statistical Analysis

#### Development of the Propensity Score

The PS is defined as the conditional probability of assignment to an intervention given a set of observed, pre-treatment variables (Rosenbaum and Rubin 1983). Moreover, the PS is a balancing score which can be used to achieve a balanced distribution for the observed covariates of the treated and control group (Austin 2011). The PS was estimated in a univariate logistic regression function for the intervention groups. Here, MST is considered the treated group (coded as 1), and FFT the comparison group (coded as 0). This is because, according to the RNR model, adolescents should be assigned to FFT unless there are serious indications to assign an adolescent to MST (Oudhof et al. 2009). The observed pre-treatment variables of adolescents are the independent variables added to the model (Austin 2011; D'Agostino Jr 1998; Thoemmes and Kim 2011). These variables, the potential confounders, were selected for the PS model based on clinical knowledge and their expected relation to at least the outcome, and possibly to the treatment itself (Brookhart et al. 2006; Stuart 2010).

#### Weighting by the Propensity Score

The PS was applied by weighting groups by the odds of their estimated PS score (Stuart 2010). Weighting by their odds was preferred because there were more treated MST cases than control FFT cases and the interest lies in the average treatment effect in the treated (ATT) rather than the average treatment effect (ATE; Stuart 2010). The ATT is the treatment effect in the adolescents who were actually treated with MST., iIn other words, treatment outcomes for adolescents who received MST are compared with outcome s effects that would have been found if the same adolescents had received FFT (Harder et al. 2010; Stuart 2010). In contrast, the ATE is the difference between the outcomes if the entire patient group had been treated with MST and the outcome if all had been treated with FFT.

For the estimation of the ATT, the MST group wasweighted with 1, while the FFT group was weighted with the PS score divided by one minus the PS score. The PS scores that showed no overlap in the treatment groups were removed. Though this restricts the generalizability of the results to cases for which overlap is present, removing cases without overlap allows for more precisely balancing the treatment arms (Harder et al. 2010).

#### Missing Indicator Approach

The baseline covariates in the dataset of 697 adolescents who completed either FFT or MST had missing values. To manage these missing values, a missing indicator approach was used while estimating the PS (Cham and West 2016; D’Agostino Jr. et al. 2001; Harder et al. 2010; Rosenbaum and Rubin 1984; West et al. 2014). This method can be theoretically justified and works well to balance observed and missing value patterns across treatment groups without removing cases from the analysis (Cham and West 2016; Harder et al. 2010; Rosenbaum and Rubin 1984). In applying this method, the covariate and a missing indicator for this covariate were included in the PS estimation, coded 1 if there was a missing value for the covariate and 0 if not (D’Agostino Jr. et al. 2001; Haviland et al. 2007; Rosenbaum 2010). This method enables the use of all cases and balances observed values in the covariates, as well as the missing patterns of these covariates. After PS estimation, balance was assessed for the missing indicators and covariates without missing value substitution. In estimating treatment effects, the missing value substitution was also removed and missing indicators were not taken into account in estimating treatment effects (Haviland et al. 2007; Rosenbaum 2010).

#### Balance Assessment

An important step in applying the PS is to assess the balance of the observed covariates between the two treatment arms (Stuart 2010). Balance was evaluated for the covariate without missing value substitution and for the missing indicators of the covariates (Harder et al. 2010; Haviland et al. 2007). Balance is achieved when the distribution of the baseline covariates is similar for the two interventions. Balance was assessed with the standardized bias which is independent of the sample size of the study. It was calculated by dividing the difference of the means of the covariates between the treated (MST) and comparison (FFT) group by the standard deviation of the treated group (i.e. MST; Austin 2009; Austin 2011; Harder et al. 2010; Stuart 2010; West et al. 2014). For the categorical covariates, the standardized bias was estimated per level (Harder et al. 2010).

The standardized bias was assessed before and after applying the PS to determine whether balance was achieved. The balance of the baseline covariates and missing indicators was assessed in the weighted sample. As a rule of thumb, it was assumed that balance was achieved when the standardized bias was less than .25 (Harder et al. 2010; Ho et al. 2007; West et al. 2014). The standardized bias of all covariates was carefully evaluated in addition to the balance of important, prognostic covariates (Ho et al. 2007).

In addition to the standardized bias, the variance ratio and the five-number-summary of the continuous covariates were assessed to determine whether these distributions were similar in higher order moments (Austin 2009). The distributions of the estimated variances are assumed to follow an F-distribution (Austin 2009). The 2.5th and 97.5th percentiles can serve as a guide as to which variance ratios are tested to be equal between the treatment groups (Austin 2009). The five-number summaries should also be used as a qualitative assessment because there is no method to test the similarity of these summaries between treatment groups (Austin 2009).

#### Analysis of Treatment Effect

Regression analysis was used to estimate treatment effect estimates in the weighted sample. The treatment effect on the primary outcome measure was estimated with an OLS regression on the outcome and the treatment indicator as an independent variable. The effect of interventions on the secondary outcome measures was analyzed with logistic regression analyses. The results were used to calculate average risk differences and risk ratios, as these measures are collapsible among subgroups, in contrast to odds ratios (Goossens et al. 2015). These measures were estimated using ordinary cross tabs of the outcomes and treatment indicators in the weighted sample. For example, for the outcome ‘living at home after treatment’, the risk ratio was estimated as the probability of living at home after MST divided by the probability of living at home after FFT. The risk difference is the difference between these probabilities, estimated as the probability of living at home after MST minus the probability of living at home after FFT. For ‘engaged in school or work’ and ‘new police contacts’, the probability of being engaged in school or work and of having had police contact during treatment were looked at. The 95% confidence intervals of the final treatment effects were estimated using simple bootstrapping (Austin and Small 2014). In total, 5000 bootstrap samples were drawn from the weighted sample and in each bootstrapped sample, treatment effects were estimated. The 95% interval was defined using a nonparametric percentile-based approach (Austin and Small 2014).

#### Subgroup Effects

Finally, within the study sample, analyses were repeated for the subsamples of youth who had a court order (246 adolescents assigned to MST; 71 FFT) and youth who did not have a court order (168 MST; 202 FFT). Within each subsample, again the balance between the treatment arms was assessed and then the PS was applied by weighting groups by the odds of the estimated PS score (Green and Stuart 2014).

The analyses were performed with IBM SPSS for Windows, version 22 (IBM Corp 2013) and Microsoft Excel (2013). The 95% confidence intervals were bootstrapped in Stata 12 (StataCorp 2011).

## Results

This section first describes the sample characteristics, then the balance in the covariates, and finally the treatment effect for respectively all adolescents in the study sample (*N* = 697), the subsample of adolescents without a court order (*n* = 370), and the subsample of adolescents with a court order (*n* = 317).

### Study Sample: All Adolescents

Within the study sample of 697 adolescents, 422 completed MST and 275 completed FFT. Of the adolescents who completed MST, 67.2% were male and 83.4% were born in the Netherlands. For FFT, 53.6% of the adolescents were male and 95.8% were born in the Netherlands (see Table 1). Comparing the treatment groups on baseline characteristics showed substantial differences in internalizing, externalizing, and total behavioral problems reported by the adolescents. Furthermore, the treatment groups differed regarding gender, country of birth, the adolescent’s living situation, level of education, previous treatment, engagement in school or work, previous court order, previous police contact, and country of birth and employment status of the primary caregiver (Table 1).

#### Balance Assessment

Before the PS application, balance was assessed in all measured baseline characteristics (see Table 1). The largest imbalances were found for internalizing problems reported on the YSR, total behavioral problems measured with the YSR, gender, previous court order, and having had police contact before treatment,. The standardized bias of these baseline variables was higher than the accepted .25 (Table 1).

After weighting, balance for all of the covariates was found when the PS model contained all covariates except for the total score of behavioral problems measured by the CBCL (Table 1). Balance was inspected in the sample with overlapping PS scores. As a result, 8 MST and 12 FFT cases were removed from the resulting sample. As Table 1 shows, values for the standardized bias after PS application are all lower than .25. The values of the standardized bias for the missing indicator variables were also lower than .25 (Table III in Online Supplemental Material shows standardized bias for missing indicators before and after applying the PS).

Table 2 shows the variance ratio and five-number summaries of the continuous variables as additional measures for inspecting balance. In the weighted sample, the 2.5th and 97.5th percentiles are .78 and 1.22. The estimated variance ratios are within these boundaries, and thus equality between treatment groups using this measure can be assumed. Moreover, the five-number summaries are also roughly equal in the PS weighted sample (Table 2).

**Table 2:** Variance ratio and 5-number summary of continuous covariates after PS application in full sample (*N* = 697)

			Variance ratio‡	Minimum	25th percentile	Median	75th percentile	Maximum
Age		FFT	0.79	12.10	14.76	15.95	16.79	20.39
		MST		11.07	14.80	15.83	16.72	18.34
CBCL	Internalizing problems	FFT	0.96	33.00	55.00	61.00	68.00	88.00
		MST		33.00	55.00	62.00	69.00	82.00
	Externalizing problems	FFT	0.92	34.00	61.00	70.00	74.00	92.00
		MST		34.00	63.00	69.00	75.00	88.00
	Total behavioral problems	FFT	0.87	24.00	60.00	68.28	71.00	85.00
		MST		27.00	60.00	67.00	72.00	83.00
YSR	Internalizing problems	FFT	0.97	30.00	44.00	54.00	61.00	83.00
		MST		27.00	44.00	50.00	58.00	85.00
	Externalizing problems	FFT	1.02	29.00	52.00	59.00	66.00	80.00
		MST		29.00	51.00	58.00	66.00	93.00
	Total behavioral problems	FFT	1.06	28.00	47.00	56.00	62.00	77.00
		MST		26.00	46.00	54.00	62.00	82.00
Parenting stress		FFT	1.21	−1.40	0.61	1.98	3.34	7.78
		MST		−1.52	0.45	1.92	3.42	8.95

‡ In the weighted sample the 2.5th and 97.5th percentiles of the F-distribution are 0.78 and 1.22 respectively

CBCL, Child Behavior Checklist, YSR, Youth Self Report, PS, propensity score

#### Analysis of Treatment Effect

After assessing the balance, the effectiveness of MST and FFT was compared in the outcome model. Table 3 shows no difference in externalizing problem behavior, with a small effect size of *d* = 0.01 and *d* = 0.03, on the CBCL and the YSR, respectively. The risk ratios (RR) and risk differences (RD) of the secondary outcomes showed no differences between MST and FFT for the proportion of youth living at home and having had police contact (Table 3). However, a significantly higher proportion of adolescents who had completed MST were engaged in school or work after treatment.

**Table 3:** Comparing MST with FFT average treatment effects of the treated

All adolescents:	Study sample (*N* = 697)			
	B†	95% CI		
Externalizing problem behavior CBCL	0.14	−3.23, 3.49		
Externalizing problem behavior YSR	−0.29	−2.45, 1.90		
	RR	95% CI	RD	95% CI
Police contact during treatment	1.61	0.98, 3.08	0.10	−0.01, 0.19
Living at home after treatment	0.98	0.96, 1.01	−0.02	−0.04, 0.01
Engaged in school or work after treatment	1.27**	1.06, 1.57	0.19*	0.05, 0.33
Youth without a court order:	Study sample (*n* = 370)			
	B‡	95% CI		
Externalizing problem behavior CBCL	−3.24*	−5.97, −0.39		
Externalizing problem behavior YSR	−3.33*	−5.81, −0.86		
	RR	95% CI	RD	95% CI
Police contact during treatment	1.20	0.72, 2.77	0.05	−0.10, 0.20
Living at home after treatment	0.97	0.94, 1.01	−0.03	−0.06, 0.01
Engaged in school or work after treatment	1.09	0.94, 1.31	0.07	−0.05, 0.21
Youth with a court order:	Study sample (*n* = 317)			
	B	95% CI		
Externalizing problem behavior CBCL	*Balance not achieved ‖*			
Externalizing problem behavior YSR				
	RR	95% CI	RD	95% CI
Police contact during treatment	*Balance not achieved ‖*			
Living at home after treatment				
*Engaged in school or work after treatment*				

* Confidence interval does not contain 0, ** Confidence interval does not contain 1, † Model constant in weighted sample after applying the PS, CBCL, 61.62, YSR, 54.42, ‡ Model constant in weighted sample after applying the PS, CBCL, 66.98, YSR, 58.47, ‖ Balance was not achieved, therefore the differential effectiveness of FFT and MST could not be estimated

MST, Multisystemic Therapy, FFT, Functional Family Therapy, CI, confidence interval, RD, relative difference, RR, relative risk

### Subsample: Youth without a Court Order

Of the 697 adolescents in the study sample, 370 (168 MST; 202 FFT) had no court order before receiving the intervention. Of the adolescents who had completed MST, 61.5% were male and 90.3% were born in the Netherlands. For FFT, 52.3% of the adolescents were male and 97.4% were born in the Netherlands (for an extensive comparison of the treatment arms, see Table IV in Online Supplemental Material). Comparing the treatment groups within this subsample on baseline characteristics showed significant differences in age, externalizing and total behavioral problems measured with the CBCL, parenting stress, country of birth, level of education, previous treatment, engagement in school or work, and previous police contact (Table IV in Online Supplemental Material).

#### Balance Assessment

Before the PS application, the largest imbalances (i.e., standardized bias higher than the accepted .25) were found for age, externalizing problems on the CBCL, level of education, previous treatment, and having had police contact before treatment (Table IV in Online Supplemental Material). After PS application, balance was found when all covariates except for the total score of behavioral problems measured by the CBCL were selected for the PS estimation. Before inspecting balance, 11 MST and 29 FFT cases were removed for which there was no overlap on the PS scores. Except for the standardized bias of the level of education of the adolescent, values of the standardized bias after PS application were lower than .25 (Table IV in Online Supplemental Material). Values for the standardized bias for the missing indicator variables were also lower than .25 (Table V in Online Supplemental Material). The variance ratios of the continuous variables, except for parenting stress, were within the boundaries defined by the 2.5th and 97.5th percentiles of the F-distribution in the weighted sample. Thus, except for parenting stress, balance can be assumed given these values (Table VI in Online Supplemental Material). The five-number summaries show roughly equally distributed continuous variables between the treatment groups (Table VI in Online Supplemental Material).

#### Analysis of Treatment Effect

In the subsample of adolescents without a court order, MST and FFT differed significantly in terms of externalizing problem behavior. Multisystemic Therapy resulted in lower scores on externalizing problem behavior than FFT, with a medium effect size of *d* = 0.32 and *d* = 0.34, respectively. The differences (RR and RD) between MST and FFT on the three secondary outcomes were insignificant (Table 3).

### Subsample: Youth with a Court Order

In total, 317 (246 MST; 71 FFT) of the 697 adolescents in the study sample had a court order before starting treatment. Of the adolescents who had completed MST, 70.4% were male and 78.2% were born in the Netherlands, while for FFT, 56.1% of the adolescents were male and 91% were born in the Netherlands (for an extensive comparison of the treatment arms, see Table VII in Online Supplemental Material). Multisystemic Therapy and FFT showed significant differences in terms of age, externalizing behavioral problems measured with the CBCL, internalizing problems measured with the YSR, gender, relation with father, and employment status of the primary caregiver at the baseline (Table VII in Online Supplemental Material).

#### Balance Assessment

Before the PS application, the standardized bias was higher than the accepted .25 for age, externalizing problem behavior on the CBCL, internalizing problems on the YSR, gender, relation with father, and employment status of the primary caregiver (Table VII in Online Supplemental Material). After PS application, balance was not achieved using different PS estimations. Either there were some variables with a standardized bias higher than .25, or there were numerous variables with a standardized bias just below.25. Furthermore, if balance was assessed in the sample with overlapping scores on the PS, roughly 60–80 MST cases had to be removed each time when testing various PS estimations. This indicates that the sample of adolescents assigned to MST could not be balanced to the sample of adolescents assigned to FFT (West et al. 2014).

#### Analysis of Treatment Effect

Because there was not confidence in assuming balance was achieved in this subsample of youth with a court order, the effectiveness could not be estimated without ensuring the control of allocation bias.

## Discussion

Using the PS method to control for the non-random assignment of adolescents to either MST or FFT, this study compared these two interventions on their effectiveness in the Netherlands. In the study sample, target populations were balanced and no differences between the interventions were found regarding externalizing problem behavior. Some additional results were found: adolescents assigned to MST were more often engaged in school or work after treatment. This treatment objective likely receives greater emphasis during MST than during FFT.

In the present study, the average treatment effect of the treated was estimated and the finding suggests that adolescents who receive MST may display the same treatment effects if they would have received FFT. This treatment effect, however, is only applicable for adolescents and their sample characteristics for whom there were outcome measurements after treatment. Finding only a few differences when comparing the effectiveness of MST and FFT in the overall study sample is in accordance with previous findings by Baglivio et al. (2014).

As the present study demonstrates that adolescents with a court order — interpreted as a possible risk factor following the RNR-model (Andrews et al. 2006; van der Laan et al. 2010) — were more often assigned to MST (246 MST; 71 FFT), MST could also be expected to be more effective in this subsample. However, due to the incomparability of the FFT and MST subsamples of youth with a court order, the present study cannot confirm this. Furthermore, following the RNR model, FFT could at least be expected to be effective in the subsample of adolescents without a court order, as these adolescents would be expected to have lower risks, and, therefore, less intensive treatment would be adequate (Andrews et al. 2006; van der Laan et al. 2010). It was shown that FFT was effective, as it reduced externalizing problems from 67.08 on average (Table 1) to 61.62 on average (model constant in the weighted sample after applying the PS). However, MST was more effective in reducing externalizing problems in the subsample of youth without a court order. This may be explained by the fact that a more intensive treatment in a less severe target population is always likely to be more effective, but the question remains as to whether it is appropriate and proportional treatment. Furthermore, it could be explained by the fact that, although some risk factors were less present in the group without a court order, such as engagement in school or police contact (Table I and IV in Online Supplemental Material), this group nevertheless reported more problem behavior measured with the CBCL and the YSR (Tables I and IV in Online Supplemental Material). Another explanation may be that having or not having a court order only provides a rough indication of the risk level of an adolescent, while clinicians assign adolescents to either MST or FFT based on other risk factors as well. The RNR model thus leaves room for interpretation, or a single characteristic cannot fully represent the risk level of an adolescent. For the secondary outcomes, however, no differences were found between the interventions, though these outcomes may be highly relevant to society. This should be taken into account when interpreting the overall effectiveness of the interventions in this subgroup. Furthermore, future research could focus on the applicability and validity of a checklist based on the RNR model, for example, to support stepped care when applicable, and assign adolescents directly to more intensive interventions when needed (Krugten et al. 2016).

In addition to the effectiveness and assignment procedures of the interventions, and with stringent health care budgets, the costs of an intervention should be taken into account. If costs of a more effective intervention are higher than the costs of its alternative, it can be worthwhile to compare the interventions and their cost-effectiveness. Previous studies in the US and UK have shown MST to be cost-effective compared with alternatives like individual therapy (Cary et al. 2013; Klietz et al. 2010). The cost-benefit ratio of FFT compared to MST in the US has been shown to be in favor of FFT (Lee et al. 2012). In the Netherlands, Vermeulen et al. (2017) compared MST to treatment as usual, including FFT, and found MST to be more cost-effective. Thus, cost-effectiveness depends on the context of the study (e.g., sample or country). With regard to the current study, it would for example be beneficial to implement a cost-effectiveness analysis in the subsample of adolescents without a court order. In this subsample, MST was more effective at reducing externalizing problems than FFT. Although it is unknown what the precise costs of MST and FFT are in the Netherlands, it is expected that MST is more expensive due to the intensity of the intervention. Cost-effectiveness analysis could reveal whether additional costs for MST are worth the higher effects. Future research should focus on estimating the exact costs of MST and FFT in the Netherlands and on estimating health services use of this population to indeed estimate the cost-effectiveness. Moreover, it is of interest to determine the cost-effectiveness of intervention options when following a stepped care procedure (i.e., should youth with a lower risk be assigned to MST directly, or should a less intensive option be the first choice).

Comparing evidence-based interventions within overlapping target populations could eventually result in greater knowledge about which interventions work best for whom (Yirmiya 2010). Therefore, it is important to examine treatment through client interactions and understand and study the assignment procedure based on the RNR model in greater detail. However, given the broad range of interventions currently available, it seems even more necessary to study practice elements or program elements of interventions to determine overlapping, effective elements (Chorpita and Daleiden 2009; Evenboer et al. 2012; Lee et al. 2014). Furthermore, it would be of interest to compare the long-term effects of MST and FFT to find out whether their comparative effectiveness changes over time.

This effectiveness study also shows that using clinical practice data, like ROM data, is worthwhile for evaluating treatments. It increases both the external validity of the study and the clinical utility, because data was gathered in regular clinical practice and sample selection bias is less present (Hodgson et al. 2007). The current study shows that the PS method is a useful and important method for using these data (West et al. 2014). It is, however, relevant to evaluate the chosen treatment outcomes in light of the selected dataset. The current study selected data from the Viersprong and not from other youth care institutions. Moreover, of the data available, a sample was selected for which there was an outcome measure after treatment. The study sample within which the comparative effectiveness was studied consisted of adolescents with overall less risk factors (i.e., less reported court orders, see Table I and II in Online Supplemental Material) compared to the group for which no data was available after treatment, which could in turn result in less differences between interventions because this group might have shown better results overall. Thus, although clinical practice data were used, the findings can only be generalized to the selected group of adolescents and the findings should be interpreted in light of this sample selection. On the one hand, this study sample is likely larger and has less sample selection bias compared to data from randomized clinical trials (RCTs). On the other hand, using observational data still merits reflection on the generalizability of the findings and evaluation given the selections, regardless of the study design (Stuart et al. 2011). Furthermore, partial replication of a previous study (Baglivio et al. 2014) supports prior evidence and shows that the results are robust across different clinical settings and study designs (Duncan et al. 2014).

Because our study is an effectiveness study and not an efficacy study, the interventions were studied as delivered in daily clinical practice as opposed to under highly controlled circumstances. In an efficacy study, interventions are more standardized and studied in rather homogeneous populations (Glasgow et al. 2003; Nordon et al. 2016; Singal et al. 2014). Though MST and FFT are both monitored by a quality system, follow detailed protocols, and require therapists to have completed specific training, the population treated, the duration and intensity of the treatment, and adherence of therapists to the treatment protocol may vary as a result of adapting the treatment to ever-changing circumstances in daily clinical practice. We chose not to control for such variations within and differences between the interventions, because then our study would no longer fully represent the effectiveness of the services as provided. Future research could be of interest to define treatment variables that should be reckoned with in clinical practice, such as specific or common program or practice elements that are important to obtain favorable treatment outcomes.

Despite the clinical relevance and use of this study, some limitations merit reflection. First, although a wide range of initial differences between adolescents in the treatment arms were controlled for, there could still be differences that were unmeasured and thus not controlled for. For example, the quality of life of the adolescent was not measured. This could have led to hidden biases in the presented results (Rosenbaum 1991; Shadish 2013). Second, though a response rate of about 40% is common when using clinical practice data from ROM in the Netherlands that are not gathered for specific research purposes, there were a number of families who did not complete the CBCL at the end of the treatment. When comparing adolescents who did and did not complete this primary outcome measure, there were differences within the MST and FFT group. As a result, the external validity of this study is not optimal because the effect of the treatments in the group with missing data could not be established. Third, we did not use a control group of adolescents without any treatment. It would, however, be helpful to include a reference treatment when policy makers have to decide on the use of these two evidence-based interventions. Fourth, although the chosen method was thoroughly considered, and all assumptions were checked, the choice of methods could influence the outcomes. There could, for example, be other estimation methods (e.g., matching with the PS or stratification using the PS), which arrive even closer to the true effect (Cham and West 2016; Harder et al. 2010). Even more, using different approaches can help reducing uncertainty surrounding outcomes. Finally, the subgroup that was chosen to indicate risk level according to the RNR model was based on having a court order or not, but other demographic characteristics (in combination) could have been used to study subgroups as well, such as living situation or education level.

In conclusion, the current study found few differences in the relative effectiveness of MST and FFT. This paper also stresses the necessity of investigating effects within subgroups of adolescents, as conclusions can change when looking at specific subgroups. Though RCTs are considered to be most effective for evaluating treatment options, using clinical practice data is certainly a viable alternative when carefully applied. By thoroughly controlling for treatment selection, the approach even enhances external validity because sample selection is less present than in RCTs (Stuart et al. 2011).

## Electronic supplementary material

ESM 1 (PDF 649 kb)
