# Supplementary material for: Multisystemic Therapy and Functional Family Therapy Compared on their Effectiveness Using the Propensity Score Method
Source: J Abnorm Child Psychol. 2018 Jan 9;46(5):1037–50. doi: 10.1007/s10802-017-0392-4 (PMC6010495; doi:10.1007/s10802-017-0392-4)
Supplement: Supplementary file 1 — (PDF 649 kb) [file 10802_2017_392_MOESM1_ESM.pdf]

Table I: Excluded versus included adolescents due to missing outcome measure after treatment (FFT)

**Variable**		**Excluded**	***(n = 365)***		**Included**	***(n = 275)***		**Test statistic**
Continuous variables		***Mean***	***SD***	***N***	***Mean***	***SD***	***N***	***T-test***
Age		15.95	1.65	365	15.9	1.59	275	0.70
CBCL	Internalizing problems	62.5	10.45	268	62.51	9.26	263	-0.02
Primary outcome	Externalizing problems	65.88	10.49	268	67.08	9.57	263	-1.37
	Total behavioral problems	65.17	9.93	268	66.04	8.61	263	-1.08
YSR	Internalizing problems	53.54	10.78	228	54.79	11.31	246	-1.24
	Externalizing problems	58.01	10.28	228	59.27	9.73	246	-1.37
	Total behavioral problems	65.05	9.53	228	57.35	9.78	246	-1.46
Parenting stress		2	1.92	248	1.97	1.78	258	0.21
Categorical variables		***%***		***N***	***%***		***N***	***Chi-Square statistic***
Gender	Male	50.1		183	53.6		141	0.74
	Female	49.9		182	46.4		122	
Country of birth	Netherlands	90.1		301	95.8		253	7.17*
	Western country	3.3		11	1.1		3	
	Non-Western country	6.6		22	3.0		8	
Living situation adolescent	Together with one parent	45.0		154	36.1		97	9.20**
	Together with multiple parents	48.8		167	60.6		163	
	Other	6.1		21	3.3		9	
Living situation adolescent	Lived not at home	2.1		7	0.8		2	1.76
Secondary outcome	Lived at home	97.9		326	99.2		260	
Level of education	None	9.2		30	7.1		19	1.77
	Primary education	4.0		13	3.7		10	
	Lower secondary education	52.5		171	54.5		146	
	Higher secondary education	34.0		111	34.7		93	
	Higher education	0.3		1	0.0		0	
Previous treatment	Absent	10.4		35	9.8		26	0.06

	Present	89.6		302	90.2		240	
Engagement in school or work	Absent	20.3		64	14.5		37	3.34
Secondary outcome	Present	79.7		251	85.5		219	
Court order	No	67.0		229	74.0		202	7.76*
	Civil	18.7		64	10.6		29	
	Criminal	14.3		49	15.4		42	
Police contacts during treatment	Absent	73.4		240	66.9		176	2.94
Secondary outcome	Present	26.6		87	33.1		87	
Relation father	Absent	10.2		33	6.8		17	2.04
	Present	89.8		292	93.2		234	
Relation mother	Absent	1.2		4	0.4		1	1.07
	Present	98.8		330	99.6		249	
Relation siblings	Absent	9.2		29	7.6		18	0.42
	Present	90.8		287	92.4		218	
Relation peers	Absent	0.6		2	0.0		0	1.51
	Present	99.4		330	100.0		249	
Country of birth primary caregiver	the Netherlands	85.6		274	88.5		232	3.04
	Western country	3.1		10	4.2		11	
	Non-Western country	11.3		36	7.3		19	
Level of education primary caregiver	None	3.8		11	1.2		3	5.47
	Primary education	5.2		15	4.1		10	
	Lower secondary education	31.4		90	27.9		68	
	Higher secondary education	41.5		119	45.5		111	
	Higher education	18.1		52	21.3		52	
Employment primary caregiver	Employed	66.0		214	71.8		186	2.22
	Unemployed	34.0		110	28.2		73	
Partner primary caregiver	Absent	25.3		80	21.7		55	0.99

	Present	74.7		236	78.3		198	

* p < .05, ** p < .01, *** p < .001
NOTE: Values depict the mean values and standard deviations. Except for age and parenting stress all other scores are standardized T-scores, having a mean of 50 and a standard deviation of 10. For NOSI-R and parenting stress, normed z-scores are displayed.
FFT, Functional Family Therapy, CBCL, Child Behavior Checklist, YSR, Youth Self Report, SD, standard deviation

Table II: Excluded versus included adolescents due to missing outcome measure after treatment (MST)

**Variable**		**Excluded**	***(n = 652)***		**Included**	***(n = 422)***		**Test statistic**
Continuous variables		***Mean***	***SD***	***N***	***Mean***	***SD***	***N***	***T-test***
Age		15.65	1.43	652	15.67	1.35	422	-0.22
CBCL	Internalizing problems	61.42	9.71	485	61.04	9.68	409	0.58
Primary outcome	Externalizing problems	68.09	10.13	485	68.29	10.06	409	-0.31
	Total behavioral problems	65.45	9.29	485	65.32	9.76	409	0.19
YSR	Internalizing problems	51.58	11.19	431	50.78	11.5	356	0.99
	Externalizing problems	57.2	10.59	431	57.54	10.87	356	-0.45
	Total behavioral problems	53.4	10.91	431	53.59	11.02	356	-0.24
Parenting stress		2.09	2.01	425	2.06	2.07	397	0.21
Categorical variables		***%***		***N***	***%***		***N***	***Chi-Square statistic***
Gender	Male	68.9		447	67.2		275	0.31
	Female	31.1		202	32.8		134	
Country of birth	Netherlands	73.7		468	83.4		341	20.32***
	Western country	3.3		21	4.6		19	
	Non-Western country	23.0		146	12.0		49	
Living situation adolescent	Together with one parent	53.5		345	42.9		179	13.09***
	Together with multiple parents	43.1		278	51.1		213	
	Other	3.4		22	6.0		25	
Living situation adolescent	Lived not at home	0.9		6	2.9		12	5.76*
Secondary outcome	Lived at home	99.1		631	97.1		400	
Level of education	None	14.8		93	13.7		56	2.07
	Primary education	3.8		24	2.7		11	

	Lower secondary education	64.3		404	66.8		274	
	Higher secondary education	16.9		106	16.8		69	
	Higher education	0.2		1	0.0		0	
Previous treatment	Absent	7.6		49	5.5		23	1.75
	Present	92.4		597	94.5		395	
Engagement in school or work	Absent	31.2		193	22.6		91	8.99**
Secondary outcome	Present	68.8		426	77.4		312	
Court order	No	30.6		197	40.6		168	13.10**
	Civil	40.1		258	30.9		128	
	Criminal	29.2		188	28.5		118	
Police contacts during treatment	Absent	46.3		285	50.8		198	1.87
Secondary outcome	Present	53.7		330	49.2		192	
Relation father	Absent	13.1		81	9.2		37	3.51
	Present	86.9		539	90.8		364	
Relation mother	Absent	1.0		6	0.7		3	0.13
	Present	99.0		623	99.3		402	
Relation siblings	Absent	7.4		45	6.0		23	0.74
	Present	92.6		563	94.0		361	
Relation peers	Absent	1.1		7	1.3		5	0.04
	Present	98.9		615	98.7		393	
Country of birth primary caregiver	the Netherlands	59.3		369	79.3		325	52.10***
	Western country	4.2		26	4.9		20	
	Non-Western country	36.5		227	15.9		65	
Level of education primary caregiver	None	8.7		53	3.0		12	40.86***
	Primary education	18.1		110	8.0		32	
	Lower secondary education	29.7		181	31.3		126	
	Higher secondary education	31.0		189	39.1		157	
	Higher education	12.5		76	18.7		75	

Employment primary caregiver	Employed	47.8		301	61.9		253	19.75***
	Unemployed	52.2		329	38.1		156	
Partner primary caregiver	Absent	32.7		196	23.9		94	8.89**
	Present	67.3		403	76.1		299	

* p < .05, ** p < .01, *** p < .001

NOTE: Values depict the mean values and standard deviations. Except for age and parenting stress all other scores are standardized T-scores, having a mean of 50 and a standard deviation of 10. For NOSI-R and parenting stress, normed z-scores are displayed.

MST, Multisystemic Therapy, CBCL, Child Behavior Checklist, YSR, Youth Self Report, SD, standard deviation

Table III: Standardized bias of missing indicators in full sample (N = 697)

Missing indicators§			***Before PS application***	***After PS application***
CBCL	Internalizing problems	Missing	0.07	0.07
		Not missing	0.07	0.07
	Externalizing problems	Missing	0.07	0.07
		Not missing	0.07	0.07
	Total behavioral problems	Missing	0.07	0.07
		Not missing	0.07	0.07
YSR	Internalizing problems	Missing	0.14	0.11
		Not missing	0.14	0.11
	Externalizing problems	Missing	0.14	0.11
		Not missing	0.14	0.11
	Total behavioral problems	Missing	0.14	0.11
		Not missing	0.14	0.11
Parenting stress		Missing	0.01	0.06
		Not missing	0.01	0.06
Gender		Missing	0.07	0.07
		Not missing	0.07	0.07
Country of birth		Missing	0.05	0.04
		Not missing	0.05	0.04
Living situation adolescent		Missing	0.09	0.02
		Not missing	0.09	0.02

Living situation adolescent		Missing	0.16	0.04
Secondary outcome		Not missing	0.16	0.04
Level of education		Missing	0.02	0.07
		Not missing	0.02	0.07
Previous treatment		Missing	0.24	0.05
		Not missing	0.24	0.05
Engagement in school or work		Missing	0.12	0.09
Secondary outcome		Not missing	0.12	0.09
Court order		Missing	0.09	0.01
		Not missing	0.09	0.01
Police contacts during treatment		Missing	0.12	0.03
Secondary outcome		Not missing	0.12	0.03
Relation father		Missing	0.17	0.03
		Not missing	0.17	0.03
Relation mother		Missing	0.26	0.03
		Not missing	0.26	0.03
Relation siblings		Missing	0.18	0.09
		Not missing	0.18	0.09
Relation peers		Missing	0.16	0.05
		Not missing	0.16	0.05
Country of birth primary caregiver		Missing	0.11	0.20
		Not missing	0.11	0.20
Level of education primary caregiver		Missing	0.31	0.01
		Not missing	0.31	0.01
Employment primary caregiver		Missing	0.16	0.05
		Not missing	0.16	0.05
Partner primary caregiver		Missing	0.05	0.11
		Not missing	0.05	0.11

§ No missing values were present for the variable ‘Age’, thus the missing indicator was not needed.
CBCL, Child Behavior Checklist, YSR, Youth Self Report, PS, propensity score

Table IV: Baseline differences between adolescents assigned to FFT and MST and standardized bias for youth without a court order (n=370)

**Variable**		**FFT**	***(n = 202)***		**MST**	***(n = 168)***		**Test statistic**	**Standardized bias**	
Continuous variables		***Mean***	***SD***	***N***	***Mean***	***SD***	***N***	***T-test***	***Before PS application***	***After PS application***
Age		15.72	1.61	202	15.28	1.39	168	2.76**	0.32	0.05
CBCL	Internalizing problems	62.99	8.72	195	62.99	8.58	161	0.00	0.00	0.01
Primary outcome	Externalizing problems	68.54	8.48	195	72.2	7.87	161	-4.18***	0.47	0.04
	Total behavioral problems †	67.31	7.39	195	68.84	6.8	161	-2.02*	0.23	0.10
YSR	Internalizing problems	55.21	11.15	182	52.76	11.42	139	1.93	0.22	0.04
	Externalizing problems	59.96	8.84	182	60.73	9.91	139	-0.73	0.08	0.02
	Total behavioral problems	58.34	8.96	182	56.76	9.8	139	1.50	0.16	0.02
Parenting stress		2.23	1.71	188	2.67	2.11	161	-2.12*	0.21	0.09
Categorical variables		***%***		***N***	***%***		***N***	***Chi-Square statistic***		
Gender	Male	52.3		102	61.5		99	3.03	0.19	0.10
	Female	47.7		93	38.5		62		0.19	0.10
Country of birth	Netherlands	97.4		190	90.3		149	8.97*	0.13	0.06
	Western country	1.0		2	1.8		3		0.01	0.00
	Non-Western country	1.5		3	7.9		13		0.12	0.06
Living situation adolescent	Together with one parent	31.3		62	35.5		59	1.23	0.08	0.22
	Together with multiple parents	66.7		132	61.4		102		0.10	0.25
	Other	2.0		4	3.0		5		0.02	0.03
Living situation adolescent	Lived not at home	0.0		0	0.0		0	NA	0.00	0.00
Secondary outcome	Lived at home	100.0		194	100.0		165		0.00	0.00
Level of education	None	5.1		10	8.4		14	23.81***	0.05	0.12
	Primary education	3.6		7	3.6		6		0.00	0.06
	Lower secondary education	53.8		106	73.1		122		0.27	0.27
	Higher secondary education	37.6		74	15.0		25		0.31	0.09
Previous treatment	Absent	21.5		20	4.8		8	3.94*	0.26	0.06

	Present	78.5		73	95.2		160		0.26	0.06
Engagement in school or work	Absent	12.1		23	20.0		33	4.14*	0.20	0.06
Secondary outcome	Present	87.9		167	80.0		132		0.20	0.06
Court order	No	100.0		202	100.0		168	NA	0.00	0.00
	Civil	0.0		0	0.0		0		0.00	0.00
	Criminal	0.0		0	0.0		0		0.00	0.00
Police contacts during treatment	Absent	72.0		139	55.1		87	10.90***	0.34	0.04
Secondary outcome	Present	28.0		54	44.9		71		0.34	0.04
Relation father	Absent	8.2		15	6.1		10	0.55	0.09	0.03
	Present	91.8		169	93.9		154		0.09	0.03
Relation mother	Absent	0.6		1	0.6		1	0.00	0.01	0.00
	Present	99.4		180	99.4		165		0.01	0.00
Relation siblings	Absent	38.5		10	5.6		9	0.00	0.00	0.03
	Present	61.5		16	94.4		151		0.00	0.03
Relation peers	Absent	0.0		0	0.6		1	1.11	0.08	0.00
	Present	100.0		180	99.4		161		0.08	0.00
Country of birth primary caregiver	the Netherlands	89.5		171	88.3		144	2.96	0.02	0.09
	Western country	4.2		8	1.8		3		0.04	0.00
	Non-Western country	6.3		12	9.8		16		0.06	0.09
Level of education primary caregiver	None	1.1		2	0.6		1	1.14	0.01	0.00
	Primary education	2.2		4	3.1		5		0.01	0.03
	Lower secondary education	23.3		42	22.7		37		0.01	0.02
	Higher secondary education	50.6		91	47.2		77		0.04	0.04
	Higher education	22.8		41	26.4		43		0.04	0.02
Employment primary caregiver	Employed	69.8		132	69.1		112	0.02	0.02	0.15
	Unemployed	30.2		57	30.9		50		0.02	0.15
Partner primary caregiver	Absent	21.1		39	16.1		26	1.37	0.13	0.09
	Present	78.9		146	83.9		135		0.13	0.09

* p < .05, ** p < .01, *** p < .001, † Not selected for PS estimation.
NOTE: Values depict the mean values and standard deviations. Except for age and parenting stress all other scores are standardized T-scores, having a mean of 50 and a standard deviation of 10. For NOSI-R and parenting stress, normed z-scores are displayed.
MST, Multisystemic Therapy, FFT, Functional Family Therapy, CBCL, Child Behavior Checklist, YSR, Youth Self Report, SD, standard deviation, PS, propensity score

Table V: Standardized bias of missing indicators in sample of youth without a court order (n = 370)

Missing indicators§			***Before PS application***	***After PS application***
CBCL	Internalizing problems	Missing	0.04	0.06
		Not missing	0.04	0.06
	Externalizing problems	Missing	0.04	0.06
		Not missing	0.04	0.06
	Total behavioral problems	Missing	0.04	0.06
		Not missing	0.04	0.06
YSR	Internalizing problems	Missing	0.19	0.03
		Not missing	0.19	0.03
	Externalizing problems	Missing	0.19	0.03
		Not missing	0.19	0.03
	Total behavioral problems	Missing	0.19	0.03
		Not missing	0.19	0.03
Parenting stress		Missing	0.14	0.06
		Not missing	0.14	0.06
Gender		Missing	0.04	0.06
		Not missing	0.04	0.06
Country of birth		Missing	0.13	0.00
		Not missing	0.13	0.00
Living situation adolescent		Missing	0.07	0.06
		Not missing	0.07	0.06
Living situation adolescent		Missing	0.16	0.11
Secondary outcome		Not missing	0.16	0.11
Level of education		Missing	0.24	0.08

		Not missing	0.24	0.08
Previous treatment		Missing	0.00	0.00
		Not missing	0.00	0.00
Engagement in school or work		Missing	0.31	0.11
Secondary outcome		Not missing	0.31	0.11
Court order		Missing	0.00	0.00
		Not missing	0.00	0.00
Police contacts during treatment		Missing	0.06	0.06
Secondary outcome		Not missing	0.06	0.06
Relation father		Missing	0.43	0.00
		Not missing	0.43	0.00
Relation mother		Missing	0.85	0.06
		Not missing	0.85	0.06
Relation siblings		Missing	0.36	0.00
		Not missing	0.36	0.00
Relation peers		Missing	0.39	0.00
		Not missing	0.39	0.00
Country of birth primary caregiver		Missing	0.15	0.00
		Not missing	0.15	0.00
Level of education primary caregiver		Missing	0.46	0.00
		Not missing	0.46	0.00
Employment primary caregiver		Missing	0.15	0.10
		Not missing	0.15	0.10
Partner primary caregiver		Missing	0.21	0.03
		Not missing	0.21	0.03

§ No missing values were present for the variable ‘Age’, thus the missing indicator was not needed.
CBCL, Child Behavior Checklist, YSR, Youth Self Report, PS, propensity score

Table VI: Variance ratio and 5-number summary of continuous covariates after PS application in sample of youth without court order (n = 370)

			**Variance ratio‡**	**Minimum**	**25th percentile**	**Median**	**75th percentile**	**Maximum**

Age		FFT	0.83	12.10	14.25	15.18	16.08	20.39
		MST		11.07	14.54	15.29	16.39	17.88
CBCL	Internalizing problems	FFT	0.96	39.00	57.00	65.00	69.00	83.00
		MST		34.00	57.75	64.00	70.00	81.00
	Externalizing problems	FFT	1.09	43.00	69.00	72.00	76.00	92.00
		MST		46.00	68.75	73.50	77.00	88.00
	Total behavioral problems	FFT	1.03	44.00	65.00	70.00	74.00	84.00
		MST		50.00	65.00	70.00	74.00	82.00
YSR	Internalizing problems	FFT	1.17	30.00	45.00	54.00	60.54	83.00
		MST		30.00	46.00	53.00	62.00	81.00
	Externalizing problems	FFT	1.25	37.00	55.00	60.96	68.01	80.00
		MST		29.00	54.00	61.00	68.00	88.00
	Total behavioral problems	FFT	1.18	34.00	51.00	56.06	63.20	77.00
		MST		26.00	50.00	56.00	64.00	82.00
Parenting stress		FFT	1.40	-1.16	1.23	2.62	3.54	7.78
		MST		-1.38	1.20	2.59	4.13	8.95

‡ In the weighted sample the 2.5th and 97.5th percentiles of the F-distribution are 0.63 and 1.37 respectively.
CBCL, Child Behavior Checklist, YSR, Youth Self Report, PS, propensity score

Table VII: Baseline differences between adolescents assigned to FFT and MST and standardized bias for youth with court order (n=317)

**Variable**		**FFT**	***(n = 71)***		**MST**	***(n = 246)***		**Test statistic**	**Standardized bias**	
Continuous variables		***Mean***	***SD***	***N***	***Mean***	***SD***	***N***	***T-test***	***Before PS application***	***After PS application***
Age		16.4	1.42	71	15.94	1.26	246	2.58**	0.36	NA
CBCL	Internalizing problems	60.77	10.25	66	59.84	10.14	240	0.66	0.09	NA
Primary outcome	Externalizing problems	62.85	11.25	66	65.78	10.52	240	-1.97*	0.28	NA
	Total behavioral problems	62.18	10.73	66	63.09	10.7	240	-0.61	0.09	NA
YSR	Internalizing problems	53.16	11.64	62	49.59	11.52	209	2.14*	0.31	NA
	Externalizing problems	57.21	11.85	62	55.55	11.01	209	1.02	0.15	NA
	Total behavioral problems	54.29	11.52	62	51.27	11.38	209	1.65	0.24	NA

Parentingl stress		1.24	1.82	68	1.68	1.93	228	-1.66	0.23	NA
Categorical variables		*%*		***N***	*%*		***N***	***Chi-Square statistic***		
Gender	Male	56.1		37	70.4		169	4.85*	0.31	NA
	Female	43.9		29	29.6		71		0.31	NA
Country of birth	Netherlands	91.0		61	78.2		186	5.92	0.18	NA
	Western country	1.5		1	6.7		16		0.07	NA
	Non-Western country	7.5		5	15.1		36		0.10	NA
Living situation adolescent	Together with one parent	47.8		33	48.6		119	0.10	0.01	NA
	Together with multiple parents	44.9		31	43.3		106		0.03	NA
	Other	7.2		5	8.2		20		0.01	NA
Living situation adolescent	Lived not at home	3.0		2	5.0		12	0.45	0.09	NA
Secondary outcome	Lived at home	97.0		64	95.0		229		0.09	NA
Level of education	None	13.0		9	17.6		42	2.90	0.05	NA
	Primary education	4.3		3	2.1		5		0.02	NA
	Lower secondary education	58.0		40	61.9		148		0.04	NA
	Higher secondary education	24.6		17	18.4		44		0.07	NA
Previous treatment	Absent	8.5		6	5.7		14	0.70	0.12	NA
	Present	91.5		65	94.3		231		0.12	NA
Engagement in school or work	Absent	21.9		14	24.8		58	0.23	0.07	NA
Secondary outcome	Present	78.1		50	75.2		176		0.07	NA
Court order	No	0.0		0	0.0		0	2.76	0.00	NA
	Civil	40.8		29	52.0		128		0.22	NA
	Criminal	59.2		42	48.0		118		0.22	NA
Police contacts during treatment	Absent	51.5		35	47.6		108	0.32	0.08	NA
Secondary outcome	Present	48.5		33	52.4		119		0.08	NA
Relation father	Absent	3.0		2	11.6		27	4.30*	0.27	NA
	Present	97.0		64	88.4		206		0.27	NA
Relation mother	Absent	0.0		0	0.9		2	0.58	0.09	NA
	Present	100.0		68	99.1		233		0.09	NA

Relation siblings	Absent	12.1		7	6.4		14	2.14	0.23	NA
	Present	87.9		51	93.6		206		0.23	NA
Relation peers	Absent	0.0		0	1.7		4	1.19	0.13	NA
	Present	100.0		68	98.3		228		0.13	NA
Country of birth primary caregiver	the Netherlands	85.5		59	72.6		175	4.87	0.16	NA
	Western country	4.3		3	7.1		17		0.03	NA
	Non-Western country	10.1		7	20.3		49		0.13	NA
Level of education primary caregiver	None	1.6		1	4.7		11	1.88	0.03	NA
	Primary education	9.7		6	11.5		27		0.02	NA
	Lower secondary education	41.9		26	37.2		87		0.05	NA
	Higher secondary education	30.6		19	32.9		77		0.02	NA
	Higher education	16.1		10	13.7		32		0.02	NA
Employment primary caregiver	Employed	76.5		52	57.3		138	8.26**	0.39	NA
	Unemployed	23.5		16	42.7		103		0.39	NA
Partner primary caregiver	Absent	24.2		16	30.1		68	0.85	0.13	NA
	Present	75.8		50	69.9		158		0.13	NA

* $p < .05$, ** $p < .01$, *** $p < .001$

NOTE: Values depict the mean values and standard deviations. Except for age and parenting stress all other scores are standardized T-scores, having a mean of 50 and a standard deviation of 10. For NOSI-R and parenting stress, normed z-scores are displayed.

MST, Multisystemic Therapy, FFT, Functional Family Therapy, CBCL, Child Behavior Checklist, YSR, Youth Self Report, SD, standard deviation, PS, propensity score

Table VIII: Standardized bias of missing indicators in sample of youth with court order (n = 317)

Missing indicators§			***Before PS application***	***After PS application***
CBCL	Internalizing problems	Missing	0.30	NA
		Not missing	0.30	NA
	Externalizing problems	Missing	0.30	NA
		Not missing	0.30	NA
	Total behavioral problems	Missing	0.30	NA
		Not missing	0.30	NA

YSR	Internalizing problems	Missing	0.07	NA
		Not missing	0.07	NA
	Externalizing problems	Missing	0.07	NA
		Not missing	0.07	NA
	Total behavioral problems	Missing	0.07	NA
		Not missing	0.07	NA
Parenting stress		Missing	0.12	NA
		Not missing	0.12	NA
Gender		Missing	0.30	NA
		Not missing	0.30	NA
Country of birth		Missing	0.13	NA
		Not missing	0.13	NA
Living situation adolescent		Missing	0.38	NA
		Not missing	0.38	NA
Living situation adolescent		Missing	0.35	NA
Secondary outcome		Not missing	0.35	NA
Level of education		Missing	0.00	NA
		Not missing	0.00	NA
Previous treatment		Missing	0.06	NA
		Not missing	0.06	NA
Engagement in school or work		Missing	0.23	NA
Secondary outcome		Not missing	0.23	NA
Court order		Missing	0.00	NA
		Not missing	0.00	NA
Police contacts during treatment		Missing	0.13	NA
Secondary outcome		Not missing	0.13	NA
Relation father		Missing	0.08	NA
		Not missing	0.08	NA
Relation mother		Missing	0.01	NA

		Not missing	0.01	NA
Relation siblings		Missing	0.25	NA
		Not missing	0.25	NA
Relation peers		Missing	0.06	NA
		Not missing	0.06	NA
Country of birth primary caregiver		Missing	0.06	NA
		Not missing	0.06	NA
Level of education primary caregiver		Missing	0.36	NA
		Not missing	0.36	NA
Employment primary caregiver		Missing	0.16	NA
		Not missing	0.16	NA
Partner primary caregiver		Missing	0.04	NA
		Not missing	0.04	NA

§ No missing values were present for the variable ‘Age’, thus the missing indicator was not needed.
CBCL, Child Behavior Checklist, YSR, Youth Self Report, PS, propensity score
